# Effect of a plant sterol-enriched spread on biomarkers of endothelial dysfunction and low-grade inflammation in hypercholesterolaemic subjects

**DOI:** 10.1017/jns.2016.40

**Published:** 2016-12-06

**Authors:** R. T. Ras, D. Fuchs, W. P. Koppenol, C. G. Schalkwijk, A. Otten-Hofman, U. Garczarek, A. Greyling, F. Wagner, E. A. Trautwein

**Affiliations:** 1 Unilever Research and Development Vlaardingen, Vlaardingen, The Netherlands; 2 Maastricht University Medical Centre, Maastricht, The Netherlands; 3 Charité Research Organisation, Berlin, Germany

**Keywords:** Plant sterols, Low-grade inflammation, Endothelial dysfunction, Dietary interventions, CRP, C-reactive protein, FMD, flow-mediated dilation, PS, plant sterols, sE-selectin, soluble endothelial-selectin, sICAM-1, soluble intercellular adhesion molecule-1, sVCAM-1, soluble vascular cell adhesion molecule-1

## Abstract

Plant sterols (PS) lower LDL-cholesterol, an established risk factor for CHD. Endothelial dysfunction and low-grade inflammation are two important features in the development of atherosclerosis. Whether PS affect biomarkers of endothelial function and low-grade inflammation is not well studied. The aim of the present study was to investigate the effect of regular intake of PS on biomarkers of endothelial dysfunction and low-grade inflammation. In a double-blind, randomised, placebo-controlled, parallel-group study, which was primarily designed to investigate the effect of PS intake on vascular function (clinicaltrials.gov: NCT01803178), 240 hypercholesterolaemic but otherwise healthy men and women consumed a low-fat spread with added PS (3 g/d) or a placebo spread for 12 weeks. Endothelial dysfunction biomarkers (both vascular and intracellular adhesion molecules 1 and soluble endothelial-selectin) and low-grade inflammation biomarkers (C-reactive protein, serum amyloid A, IL-6, IL-8, TNF-α and soluble intercellular adhesion molecule-1) were measured using a multi-array detection system based on electrochemiluminescence technology. Biomarkers were combined using *z*-scores. Differences in changes from baseline between the PS and the placebo groups were assessed. The intake of PS did not significantly change the individual biomarkers of endothelial dysfunction and low-grade inflammation. The *z*-scores for endothelial dysfunction (−0·02; 95 % CI −0·15, 0·11) and low-grade inflammation (−0·04; 95 % CI −0·16, 0·07) were also not significantly changed after PS intake compared with placebo. In conclusion, biomarkers of endothelial dysfunction and low-grade inflammation were not affected by regular intake of 3 g/d PS for 12 weeks in hypercholesterolaemic men and women.

The LDL-cholesterol-lowering effect of foods with added plant sterols (PS) is well established^(^^1^^,^^2^^)^. Elevated blood LDL-cholesterol is causally linked to atherosclerosis and reducing LDL-cholesterol has been shown to reduce the number of CHD cases^(^^3^^)^. The direct relationship between intake of foods with added PS and CHD risk has so far not been investigated in a randomised controlled study. As for dietary interventions in general, performing a randomised controlled trial to study hard CHD endpoints with PS requiring at least 36 000 subjects being followed up for 6–10 years is rather challenging, and it can be questioned whether such a study is feasible^(^^4^^)^. As a compromise, in order to better understand the possible role of PS in CHD prevention, investigation into the effects of PS beyond LDL-cholesterol lowering, such as on endothelial dysfunction and low-grade inflammation, is warranted.

Endothelial dysfunction is one of the first clinical manifestations of atherosclerosis preceding the development of CHD. Indeed, patients with diagnosed coronary artery disease, heart failure or peripheral arterial occlusive disease overall have impaired endothelial function, and classical risk factors such as smoking, hypercholesterolaemia and hypertension are associated with endothelial dysfunction^(^^5^^–^^8^^)^. Large artery endothelial function as measured by brachial artery flow-mediated dilation (FMD) has been shown to be associated with cardiovascular risk^(^^9^^)^. We recently showed that brachial artery FMD was neither improved nor worsened with intake of a low-fat spread with added PS (3 g/d) for 12 weeks^(^^10^^)^. Other studies have shown similar findings^(^^11^^–^^13^^)^, although small improvements in FMD may be detected when analysing the data together^(^^14^^)^. Plasma endothelial cell biomarkers, such as soluble intercellular adhesion molecule-1 (sICAM-1), soluble vascular cell adhesion molecule-1 (sVCAM-1) or soluble endothelial-selectin (sE-selectin), have also been linked to CHD risk^(^^15^^)^. Few clinical studies have investigated the effect of regular PS intake on these biomarkers, mainly in metabolic syndrome patients, and overall found no effect^(^^16^^–^^18^^)^.

Another important feature in the development of atherosclerosis, and linked to endothelial dysfunction^(^^19^^,^^20^^)^, is low-grade systemic inflammation^(^^21^^,^^22^^)^. Inflammation is a common response to injury and not harmful if the system is able to return to a normal phenotype. However, when low-grade inflammation persists, this may have a negative impact on endothelial functioning, atherosclerosis and CHD risk. The anti-inflammatory effects of PS have been investigated in a few studies^(^^18^^,^^23^^)^. A recent meta-analysis summarising this evidence showed that foods with added PS (average PS dose: 2·2 g/d) overall did not significantly affect C-reactive protein (CRP)^(^^24^^)^. Also other inflammatory markers showed overall no effect. However, this meta-analysis did observe a significant dose–response relationship for the effect of PS on CRP, implying that higher PS doses (≥3 g/d) may be needed for a significant anti-inflammatory effect.

The objective of this study was to explore, in a large intervention study (*n* 240), the effect of regular PS intake (3 g/d) on plasma biomarkers of endothelial dysfunction and low-grade inflammation in hypercholesterolaemic, but otherwise healthy, individuals using a novel electrochemiluminescence-based multiplex technology. This study was part of the INVEST study (INvestigating Vascular function Effects of plant STerols) which was primarily designed to investigate the effect of a low-fat spread with added PS on FMD, and also measured arterial stiffness (pulse wave velocity and augmentation index), blood pressure, serum lipids and plasma PS concentrations^(^^10^^,^^25^^)^.

## Experimental methods

### Study population and design

This study was designed as a randomised, double-blind, placebo-controlled, 12-week, parallel study with two treatment groups. In total, 240 hypercholesterolaemic, but otherwise healthy, men and post-menopausal women (age: 40–65 years; BMI: 18–30 kg/m^2^; LDL-cholesterol: 3·4–4·9 mmol/l) were enrolled. After a 4-week run-in phase, subjects were randomised, after stratification for age, sex and screening LDL-cholesterol, across two treatment groups: low-fat spread with added PS esters (3 g/d of PS expressed as free equivalents) or low-fat placebo spread. The nutritional compositions of the fortified spread and the placebo spread were similar (i.e. 40 g fat per 100 g spread of which 10 g SFA, 10 g MUFA and 20 g PUFA). The PS mixture contained 70 % sitosterol, 14 % campesterol, 8 % sitostanol, 3 % brassicasterol and some other minor PS (sourced from BASF). The spreads were produced by Unilever Research and Development Vlaardingen. Subjects consumed two 10 g portions per d whilst maintaining their typical diet and lifestyle habits. Consumption of spreads was recorded in a diary. Opened and unopened portion packs were returned and counted to determine compliance. More details on the selection of the study population and the study design have been described elsewhere^(^^10^^)^.

This study was conducted according to the guidelines laid down in the Declaration of Helsinki and all procedures involving human subjects were approved by the ethical committee of Charité Hospital. Written informed consent was obtained from all subjects. The study was registered at clinicaltrials.gov (NCT01803178).

### Study measurements

Fasted blood samples were drawn at baseline and after 12 weeks of intervention for measuring plasma endothelial dysfunction biomarkers (sICAM-1, sVCAM-1 and sE-selectin) and low-grade inflammation biomarkers (serum amyloid A, IL-6, IL-8, TNF-α, CRP and sICAM-1). Except sE-selectin, biomarkers were measured using a multiarray detection system based on electrochemiluminescence technology (MesoScaleDiscovery, SECTOR Imager 2400) as described previously^(^^26^^)^. This system allows simultaneous, thus more efficient, analysis of different biomarkers of endothelial dysfunction and low-grade inflammation in relatively small blood samples^(^^27^^)^. Commercial ELISA kits were used for the measurement of plasma sE-selectin (Diaclone). The laboratory's intra- and interassay CV were 2·8 and 4·0 % for CRP, 2·7 and 11·6 % for serum amyloid A, 5·6 and 13·0 % for IL-6, 5·6 and 12·2 % for IL-8, 3·9 and 8·8 % for TNF-α, 2·4 and 4·9 % for sICAM-1, 2·8 ad 5·6 % for sVCAM-1 and 2·6 and 6·7 % for sE-selectin. Samples were analysed in duplicate. All samples of each subject were analysed within the same assay. Serum lipids, plasma PS concentrations, office blood pressure and vascular function measures including FMD, pulse wave velocity and augmentation index were measured as described previously^(^^10^^)^.

### Statistics

Sample size calculation was done for the primary aim of this study, i.e. FMD, as reported previously^(^^10^^)^. Data of subjects that violated the protocol, as determined in a blind review process, were excluded. Plasma concentrations of biomarkers were combined into one *z*-score for endothelial dysfunction and one for low-grade inflammation. *Z*-scores were used to combine the information from the different biomarkers, reduce the multiple testing problem, and to be more robust against perturbations of the individual biomarkers from unrelated biological and analytical sources^(^^27^^)^. Before generating *z*-scores, values of all biomarkers were log-transformed in order to generate normally distributed variables appropriate for statistical analysis. Statistical analysis was performed for each of the individual biomarkers and the two *z*-scores. An ANCOVA model was used with change from baseline as outcome, baseline values as covariate and treatment as fixed effects. Statistical analysis was performed using the statistical software package SAS version 9.4 (SAS Institute, Inc.). A two-sided *P* value <0·05 was considered statistically significant. Data are expressed as mean values and standard deviations or 95 % CI as much as possible.

## Results

At baseline, subjects (ninety female and 150 male) were aged 53·4 (sd 6·7) years and had a BMI of 25·5 (sd 2·8) kg/m^2^. Total, LDL- and HDL-cholesterol concentrations were 5·65 (sd 1·09), 3·83 (sd 0·73) and 1·39 (sd 0·46) mmol/l, respectively. More details on baseline characteristics have been presented previously^(^^10^^)^. During the study, eight out of 240 subjects dropped out. Six subjects were not weight stable, one subject used an anti-inflammatory drug (i.e. diclofenac), one subject was not fasted and three subjects had implausible values for specific biomarkers as defined during the blind review process. The data of these subjects were excluded from the per-protocol analysis. Compliance with test product intake was high (>90 %).

In the intervention group, daily intake of PS over 12 weeks significantly reduced all markers of endothelial dysfunction (Table 1). However, similar reductions were observed in the placebo group. Thus, compared with placebo, no significant effects were observed (log sE-selectin: −0·01 ng/ml (95 % CI −0·04, 0·03); log sVCAM-1: −0·02 ng/ml (95 % CI −0·07, 0·03); log sICAM-1: 0·01 ng/ml (95 % CI −0·04, 0·06)). The *z*-score for endothelial dysfunction was also not changed after PS intake *v*. placebo (log *z*-score: −0·02 (95 % CI −0·15, 0·11)).

**Table 1.** Endothelial dysfunction biomarkers in hypercholesterolaemic men and women who consumed low-fat spread with added plant sterols or placebo spread (Mean values and standard deviations or 95 % confidence intervals)

			Baseline		End of intervention		Absolute change	
Outcome	Treatment	*n*	Mean	sd	Mean	sd	Mean	95 % CI
log sE-selectin (ng/ml)	Placebo	107	4·37	0·40	4·35	0·40	−0·02	−0·05, 0·005
	PS	116	4·38	0·47	4·36	0·47	−0·03*	−0·05, −0·002
	Δ	223					−0·01	−0·04, 0·03
log sVCAM-1 (ng/ml)	Placebo	107	6·19	0·22	6·17	0·24	−0·02	−0·06, 0·01
	PS	116	6·22	0·20	6·17	0·23	−0·05*	−0·08, −0·01
	Δ	223					−0·02	−0·07, 0·03
log sICAM-1 (ng/ml)	Placebo	107	5·88	0·28	5·84	0·27	−0·05*	−0·09, −0·02
	PS	115	5·92	0·20	5·87	0·23	−0·04*	−0·08, −0·01
	Δ	222					0·01	−0·04, 0·06
*Z*-score endothelial dysfunction^†^	Placebo	107	0·01	0·72	−0·10	0·77	−0·12*	−0·22, −0·03
	PS	116	0·12	0·62	−0·04	0·66	−0·14*	−0·24, −0·05
	Δ	223					−0·02	−0·15, 0·11

sE-selectin, soluble endothelial-selectin; PS, plant sterols; sVCAM-1, soluble vascular cell adhesion molecule-1; sICAM-1, soluble intercellular adhesion molecule-1.

* Significant (*P* < 0·05).

† The *z*-score for endothelial dysfunction was calculated based on the *z*-scores of sE-selectin, sVCAM-1 and sICAM-1.

PS intake had no effect on any of the low-grade inflammation markers (Table 2). Placebo-corrected changes were −0·13 µg/ml (95 % CI −0·35, 0·10) for log CRP, −0·03 pg/ml (95 % CI −0·18, 0·12) for log IL-6, 0·03 pg/ml (95 % CI −0·05, 0·11) for log IL-8, −0·01 pg/ml (95 % CI −0·06, 0·03) for log TNF-α and −0·23 µg/ml (95 % CI −0·48, 0·02) for log serum amyloid A. In agreement, the *z*-score for low-grade inflammation was not changed after PS intake *v*. placebo (log *z*-score: −0·04 (95 % CI −0·16, 0·07)).

**Table 2.** Inflammation biomarkers in hypercholesterolaemic men and women who consumed low-fat spread with added plant sterols or placebo spread (Mean values and standard deviations or 95 % confidence intervals)

			Baseline		End of intervention		Absolute change	
Outcome	Treatment	*n*	Mean	sd	Mean	sd	Mean	95 % CI
Log CRP (μg/ml)	Placebo	107	0·02	1·13	0·10	1·25	0·10	−0·06, 0·26
	PS	115	−0·09	0·98	−0·11	0·97	−0·03	−0·19, 0·13
	Δ	222					−0·13	−0·35, 0·10
Log IL-6 (pg/ml)	Placebo	107	−0·93	0·64	−0·93	0·65	0·04	−0·06, 0·15
	PS	115	−1·05	0·51	−1·00	0·54	0·01	−0·09, 0·12
	Δ	222					−0·03	−0·18, 0·12
Log IL-8 (pg/ml)	Placebo	107	1·03	0·43	1·04	0·42	−0·0004	−0·06, 0·06
	PS	115	1·08	0·39	1·10	0·40	0·03	−0·03, 0·08
	Δ	222					0·03	−0·05, 0·11
Log TNF-α (pg/ml)	Placebo	107	0·54	0·26	0·55	0·23	0·01	−0·02, 0·04
	PS	115	0·53	0·25	0·53	0·26	−0·002	−0·03, 0·03
	Δ	222					−0·01	−0·06, 0·03
Log SAA (μg/ml)	Placebo	107	0·59	1·11	0·72	1·25	0·12	−0·06, 0·30
	PS	115	0·61	0·99	0·49	0·93	−0·11	−0·28, 0·06
	Δ	222					−0·23	−0·48, 0·02
*Z*-score low-grade inflammation^†^	Placebo	107	0·01	0·67	0·02	0·64	0·01	−0·07, 0·10
	PS	115	−0·004	0·53	−0·03	0·51	−0·03	−0·11, 0·05
	Δ	222					−0·04	−0·16, 0·07

CRP, C-reactive protein; PS, plant sterols; SAA, serum amyloid A.

† The *z*-score for low-grade inflammation was calculated based on the *z*-scores of CRP, IL-6, IL-8, TNF-α, SAA and sICAM-1.

As reported previously^(^^10^^)^, total and LDL-cholesterol concentrations were reduced significantly by 0·26 mmol/l (4·5 %) and 0·26 mmol/l (6·7 %), respectively, after intake of PS *v*. placebo. HDL-cholesterol (+0·6 %) and TAG (−2·2 %) were not significantly changed *v*. placebo. The effect of PS on FMD was +0·01 (95 % CI −0·73, 0·75) % *v*. placebo and not significant. Also arterial stiffness measures and central blood pressure were not changed after PS intake. Only office diastolic blood pressure was significantly reduced by 1·4 mmHg after PS intake *v*. placebo. As expected, plasma PS concentrations increased significantly after PS intake: plasma sitosterol by 77·9 % and campesterol by 32·6 %, *v*. placebo.

## Discussion

Biomarkers of low-grade inflammation and endothelial dysfunction are gaining increased attention in understanding CVD development and progression. As hypercholesterolaemia is linked to endothelial dysfunction^(^^28^^)^ and because PS are well known for their LDL-cholesterol-lowering effect^(^^1^^,^^2^^)^, we hypothesised that PS could beneficially affect endothelial function and inflammation. This study showed that regular intake of a low-fat spread with added PS does not change plasma concentrations of biomarkers of endothelial dysfunction and low-grade inflammation in hypercholesterolaemic men and women.

So far, a few studies have been performed that studied the effect of PS on biomarkers of endothelial dysfunction and/or low-grade inflammation. In healthy subjects, intake of PS-enriched orange juice (2 g/d PS) effectively lowered the cytokines IL-6 and IL-1b, whereas IL-8, TNF-α and plasminogen activator inhibitor-1 (PAI-1) were not significantly lowered^(^^23^^)^. In a study including fifty-eight hypercholesterolaemic subjects, rapeseed-sterol margarine (2 g/d PS) significantly reduced sE-selectin concentrations *v*. control while sVCAM-1, TNF-α and PAI-1 were not significantly reduced^(^^29^^)^. In forty-one subjects on stable statin therapy, PS or plant stanol consumption (2·5 g/d) did not change plasma concentrations of CRP, sICAM-1, sVCAM-1 or sE-selectin compared with control^(^^16^^)^. It can, however, not be ruled out that the plant stanol effect was masked by the statin therapy. In metabolic syndrome patients who are characterised by having a proinflammatory profile, intake of 2·0–2·4 g/d PS or plant stanols also did not change biomarkers of endothelial dysfunction and low-grade inflammation^(^^17^^,^^18^^)^. Significant reductions in CRP were observed in some studies^(^^30^^–^^32^^)^ but could not be confirmed in others^(^^16^^,^^33^^)^. A recently published meta-analysis found that CRP was not significantly changed (on average −0·10 mg/l (95 % CI −0·26, 0·05) for an average PS dose of 2·2 g/d), but there was some indication for a modest decrease with higher PS intakes^(^^24^^)^. Taken all the evidence together, supplementation with PS may beneficially affect biomarkers of endothelial dysfunction and low-grade inflammation, but, if present at all, effects are small.

In the present study, we used *z*-scores to increase statistical power and to reduce influences of biological variation of each individual biomarker. Nevertheless, we were not able to pick up PS effects on endothelial dysfunction and low-grade inflammation biomarkers in a hypercholesterolaemic population at a relatively high dose of 3 g/d PS. These neutral findings are in line with the observations on other vascular function measures from the present study^(^^10^^)^. Despite a small but significant correlation between individual changes in LDL-cholesterol and in endothelial function as measured by FMD, endothelial function was overall not changed^(^^10^^)^. It should be noted that, in contrast to FMD, circulating biomarkers used for endothelial function are regarded as markers derived from microvascular function and not so much from macrovascular function. Perhaps the relatively small reduction in LDL-cholesterol observed in this study (on average 7 %) was not sufficient to trigger actual changes in endothelial function. Furthermore, lowering LDL-cholesterol with statins has been shown to reduce low-grade inflammation and to improve endothelial function^(^^34^^)^, but it may well be that statins affect endothelial function also by other, cholesterol-independent, mechanisms.

We observed significant increases in plasma PS concentrations after regular intake of low-fat spread with added PS. Data from animal studies have suggested that plasma PS concentrations after feeding very high PS doses correlate with impaired endothelial-dependent vasorelaxation^(^^35^^)^, which could be a potential safety concern, when occurring in humans. The neutral findings of our study do not provide any indications that this is the case. Furthermore, we did not observe any correlation between changes in plasma PS and changes in FMD^(^^10^^)^.

Strengths of this study include the straightforward and rigid design of the study, the large sample size and the high compliance with test product intake. Some limitations of the study need to be mentioned as well. Endothelial dysfunction represents a heterogeneous set of disturbances of different physiological processes at the vascular wall (e.g. arterial stiffness, vascular tone, atherothrombosis, inflammation) and is represented by an extensive range of different biomarkers, of which only a selection is presented here. The duration of the study might have been too short to pick up substantial changes at the endothelial level. At last, the LDL-cholesterol-lowering effect observed in this study may have been too modest to actually pick up changes in endothelial function. It is unclear what may have caused this unexpected small LDL-cholesterol-lowering effect (about 7 %) for a relatively high PS dose (3 g/d).

Taken together, despite a decrease in LDL-cholesterol, biomarkers of endothelial dysfunction or low-grade inflammation were not changed after 12 weeks of PS consumption. Whether reductions in LDL-cholesterol due to PS intake would reduce CHD risk via improvements of endothelial function and low-grade inflammation requires further investigation.
